# Celiac Disease in Uzbek Children: Insights into Disease Prevalence and Clinical Characteristics in Symptomatic Pediatric Patients

**DOI:** 10.3390/diagnostics13193066

**Published:** 2023-09-27

**Authors:** Altinoy T. Kamilova, Gulnoza K. Azizova, Dimitri Poddighe, Zulkhumar E. Umarnazarova, Dilrabo A. Abdullaeva, Svetlana I. Geller, Noiba D. Azimova

**Affiliations:** 1 Gastroenterology Department, Pediatric Republican Specialized Scientific-Practical Medical Center of the Ministry of Health of Republic of Uzbekistan, Tashkent 100179, Uzbekistan; okamilova@mail.ru (A.T.K.); gulnoza91@inbox.ru (G.K.A.); zulhumorumarnazarova@gmail.com (Z.E.U.); dilrabo-uzmed@mail.ru (D.A.A.); geller_svetlana@mail.ru (S.I.G.); noiba.shakhova@gmail.com (N.D.A.); 2 School of Medicine, Nazarbayev University, Astana 010000, Kazakhstan; 3 Department of Pediatrics, National Research Center for Maternal and Child Health, University Medical Center, Astana 010000, Kazakhstan

**Keywords:** celiac disease, children, Uzbekistan, Central Asia, watery diarrhea, Bristol stool scale, zonulin, pancreatic elastase, joint pain, arthralgia

## Abstract

Background: A few studies on pediatric Celiac Disease (CD) are available from Central Asia. Recent immunogenetic research has highlighted that the HLA-DQ2/8 genetic predisposition to CD as well as the dietary intake of gluten in this geographical area, are comparable to other regions of the world where CD prevalence is known to be 1% or higher. Methods: This is a prospective and cross-sectional study investigating the prevalence and clinical characteristics of CD in symptomatic children referred to the pediatric gastroenterology department of a tertiary hospital in Uzbekistan from 1 September 2021, until 31 July 2022. In addition to collecting the relevant information related to clinical manifestations and laboratory analyses from the clinical files, a specific survey was also administered to patients’ guardians. Serological, histopathological, and immunogenetic parameters specific to CD, fecal zonulin, and pancreatic elastases were assessed in CD patients. Results: The study population consisted of 206 children. Overall, almost all of them (*n* = 192; 93.2%) were referred because of gastrointestinal manifestations, which were associated with extra-gastrointestinal manifestations in most cases (*n* = 153; 74.3%); a minority (*n* = 14; 6.8%) was mainly referred due short stature and/or growth failure only. Among all of these study participants, CD was diagnosed in 11 children (5.3%). Notably, although diarrhea was similarly reported in CD and non-CD patients, watery diarrhea (type 7 according to the Bristol stool scale) was much more frequently and significantly observed in the former group. All of these CD patients showed anti-tTG IgA 10 times higher than the upper normal limit, except one child with lower serum levels of total IgA; however, all of them received a diagnostic confirmation by histopathological analysis due to the lack of EMA testing in the country. Notably, most CD children (82%) showed a Marsh III histological grading. Around half patients (54.5%) showed zonulin values above the reference range, whereas none showed insufficient levels of pancreatic elastase. However, no correlation or association between zonulin and clinical, laboratory, histopathological, and immunogenetic parameters was found. Conclusions: This study may further suggest a relevant prevalence of CD in Uzbek children, based on this partial picture emerging from symptomatic patients only. Additionally, we highlighted the prevalence of typical CD forms with watery diarrhea, which should strongly support a full diagnostic work-up for CD in the local clinical setting. The high levels of anti-tTG IgA and high Marsh grade might also lead us to speculate a significant diagnostic delay despite the classical clinical expression of CD.

## 1. Introduction

Celiac Disease (CD) is a gluten-related and immune-mediated disorder occurring in HLA-genetically susceptible individuals. Indeed, the carriage of specific HLA-DQ alleles (especially HLA-DQB1*02) is a necessary condition to develop CD, even though this is not sufficient since dietary gluten exposure and other unveiled environmental factors are needed [1]. CD is not only a gastrointestinal disorder, although the small bowel mucosa is the main target of the immunopathological process. In addition to the damage of the intestinal villi leading to partial or total atrophy of the mucosa with related malabsorption, CD is often characterized by systemic and/or extra-intestinal manifestations, which may be underscored sometimes [2,3]. Therefore, CD may be underdiagnosed or diagnosed with delay (especially in those countries where the access to medical care is more difficult for several and variable reasons), which consequently implies a preventable burden of short- and long-term morbidity (and related healthcare costs) [4,5]. This aspect is quite relevant since CD is estimated to affect approximately 1% of the pediatric general population [6]. Notably, in children, CD can have a negative effect on the growth, pubertal, and, in general, developmental process; moreover, their longer (compared to adults) life expectation increases the probability that CD-related comorbidities may arise [7,8].

Whereas CD in children has been deeply investigated in developed countries or, in general, in those countries where its prevalence is higher than in the rest of the world, few studies are available from several geographical areas, where financial and healthcare resources are more limited and/or CD is thought (in some cases, erroneously) to be infrequent. [5,6] As regards Central Asia, a recent study demonstrated that the HLA genetic predisposition to CD in these populations is comparable to European countries, as well as the dietary intake of gluten [9]. However, despite these observations, CD is much less diagnosed than in other countries with the same genetic characteristics and dietary regimens (at least in terms of gluten foods) [10].

In this study, we investigated the prevalence and clinical characteristics of CD in symptomatic children evaluated in the referral pediatric center of national relevance in Uzbekistan.

## 2. Materials and Methods

### 2.1. Study Design and Population

This is a prospective and cross-sectional study investigating the prevalence and clinical characteristics of CD in all consecutive children with clinical manifestations suggestive of CD, who were referred to the Department of Pediatric Gastroenterology of the Republican Specialized Medical Center in Tashkent (which is a tertiary hospital of national relevance in Uzbekistan) during the study period (from 1 September 2021, until 31 July 2022).

Briefly, all consecutive children referred to the department of pediatric gastroenterology by a community health center or other peripheral hospitals, because of gastrointestinal symptoms consistent with CD, were assessed according to the 2012 ESPGHAN (European Society of Pediatric Gastroenterology, Hepatology and Nutrition) guidelines for the diagnosis of CD in children [11]. A few exclusion criteria were applied for participation in this study, as follows: (i) previously diagnosed CD; (ii) exclusion of gluten from diet at the time of study enrollment; and (iii) refusal to provide informed consent to participate in this research.

This study was conducted according to international bioethical standards and was approved by the ethical committee of the RSSPMCP (Republican Specialized Scientific-Practical Medical Center of Pediatrics, approval no. IP-2021–1223, 23 August 2021). Informed written consent was obtained from children’s guardians. The research was conducted in compliance with the Declaration of Helsinki.

### 2.2. Data Collection

In addition to collecting the relevant information related to clinical manifestations and laboratory analyses from the clinical files, a specific survey was prospectively administered to all guardians in order to collect more details (including parents’ demographic information and habits, pregnancy course, birth parameters, breastfeeding/weaning, and patients’ complaints/clinical information). This questionnaire was developed locally, based on the available literature on pediatric CD [11,12]. In detail, this survey included questions to obtain more information on all the gastroenterological (abdominal pain, excessive gas formation, bloating, flatulence, diarrhea, constipation, and vomiting) and extra-intestinal complaints (growth delay, history of anemia, pubertal development, teeth defects, skin rashes, headache, joint pain, elevated liver enzymes, and oral aphthous ulcers) that can be associated with CD, in addition to some general anamnestic information regarding personal past medical history (including comorbidities) and family history (with particular regard to the presence of first-degree relatives diagnosed with CD). Moreover, during the medical assessment, the participants were also asked to report the usual consistency of their stools according to the Bristol stool chart [13].

The physical development of the children was also assessed according to World Health Organization (WHO) guidelines [14].

### 2.3. Diagnostic Work-Up

The definitive diagnosis of pediatric CD was established according to the 2012 ESPGHAN guidelines for the diagnosis of pediatric CD. A no-biopsy pathway for symptomatic children with anti-tissue transglutaminase immunoglobulin A (anti-tTG IgA) values ≥10 times the upper normal limit and positive endomysia antibodies (EMA) IgA in a second serum sample were considered [11]. However, since the EMA IgA test was not available in our country, all anti-tTG IgA-positive subjects were recommended to undergo esophago-gastro-duodenal endoscopy (EGDS) with histological examination of the duodenal mucosa. Therefore, CD diagnosis was based upon a positive celiac serology (anti-tTG IgA) along with the presence of histological Marsh grade ≥2. IgA-deficient patients were further screened by assessing the presence of anti-tTG IgG and, if positive, histological assessment of the duodenal mucosa. All patients diagnosed with CD based on this diagnostic work-up also received HLA-DQ2 and DQ8 genotyping.

Total serum IgA was measured by using an ELISA kit based on a two-step “sandwich variant” of solid-phase ELISA using monoclonal antibodies to IgA (Cat. No A-8666, Vector-BEST, Novosibirsk, Russia). In case of low IgA levels, total IgG and anti-tTg IgG were measured. Total IgG was measured by an ELISA kit based on a two-step “sandwich variant” of solid-phase ELISA using monoclonal antibodies to IgG (Cat No. A-8662, Vector-BEST, Novosibirsk, Russia). Quantitative Anti-tTG IgA (and anti-tTG IgG, if needed) determination was carried out by using an Orgentec Diagnostika GmbH ELISA kit (Cat. No. 416-5400A, ORG 540G, Mainz, Germany).

HLA genotyping was performed using the single specific primer polymerase chain reaction (SSPPCR) DQ kits DQA1*05, DQB1*02, DQA1*0301, DQB1*0302, DQA1*0505, and DQB1*0202 for detecting the DQ2.5, DQ2.2, and DQ8 haplotypes (Celiacstrip HLA DQ2DQ8 OPERON, Inmuno and Molecular Diagnostics, Caparoca, Spain).

As regards the fecal tests (fecal zonulin and fecal pancreatic elastase), these were performed only on CD patients, by enzyme-linked immunoassay (ELISA). The IDK^®^ Zonulin ELISA kit (Immundiagnostik AG kit, Bensheim, Germany) was used for the in vitro determination of zonulin family peptides in the patient’s stool. The IDK^®^ Pancreatic Elastase ELISA kit (Immundiagnostik AG kit, Bensheim, Germany) was used for the in vitro determination of human pancreatic elastase in the patient’s stool. The stool samples were frozen and stored at −80 °C before analyzing all the collected samples in a batch.

The evaluation of the characteristic histological changes of duodenal mucosa according to the Marsh–Oberhuber grading was performed by trained histopathologists. Marsh–Oberhuber grade 2 was defined as intraepithelial lymphocyte infiltration accompanied by crypt hyperplasia, and Marsh–Oberhuber grade 3 (a/b/c) was defined as partial/subtotal/total villous atrophy in addition to intraepithelial lymphocyte infiltration and crypt hyperplasia.

### 2.4. Statistical Analysis

The database was prepared by using Microsoft Excel for Mac (version 16.74, 2021). Statistical analysis was performed by using the software GraphPad Prism (version 9.3.1, 2021). Continuous variables were expressed as median (M) and interquartile range (IQR), since the distribution was not normal according to the Kolmogorov–Smirnov normality test; differences between two groups were assessed by a two-tailed Kolmogorov–Smirnov normality test. Categorical variables were expressed as absolute numbers and percentages; differences between two or among more groups were assessed by the chi-square test or Fisher’s exact test, according to the samples’ numerosity. Spearman correlation was used to assess the correlation among some laboratory values. A *p*-value < 0.05 was considered as statistically significant.

## 3. Results

### 3.1. Study Population: Demographic and Clinical Characteristics

During the study period, a total of 248 symptomatic children with complaints potentially consistent with CD were referred to our center. However, 32 children could not be recruited because their guardians refused to give consent for their participation in this study, and another 10 children did not complete the diagnostic work-up.

Therefore, the study population consisted of 206 children (age range: 1–16 years, M:F = 98:108), who were tested for anti-tTG IgA and total IgA. The main complaints observed in all these patients are summarized in Table 1. Overall, almost all of them were referred to our center because of gastrointestinal manifestations (*n* = 192; 93.2%), including unspecific dyspeptic complaints, recurrent abdominal pain, diarrhea, bloating, vomiting, and constipation. Among them, 39 children (18.9%) complained of gastrointestinal problems only, whereas the majority (*n* = 153; 74.3%) also presented extra-gastrointestinal manifestations, including skin rashes, oral aphthae, joint pain, and anemia. A minority (*n* = 14; 6.8%) was referred without any significant gastrointestinal complaint, and, in this case, these patients were mainly referred to the gastroenterologist to assess a condition of short stature and/or growth failure.

The pediatric patients referred to our center for gastroenterological consultation showed an impairment of growth parameters, overall: indeed, the average height and weight was <−1 z-score and the average BMI was <−0.5 z-score, as summarized in Table 1. Notably, whereas height impairment was not significantly different between genders, BMI was more compromised in females (−0.78 vs. −0.21; *p* = 0.0043), which is consistent with their more frequent anamnestic report of weight loss (49.1% vs. 23.5%, *p* = 0.0002) and their lower weight at the first visit (−1.66 vs. 1.30; *p* = 0.0822) compared to male children. Moreover, weight loss was more frequent in younger patients (age classes: 1–4 years and 5–8 years); conversely growth failure is more frequently reported in older patients (age classes: 9–12 years and 13–17 years), as summarized in Table 2. No progressive age-related trends or gender-related differences are clearly evident for other clinical manifestations (see Table 1 and Table 2).

### 3.2. Celiac Disease Patients: Clinical Characteristics and Diagnostic Work-Up

Among the 206 study participants, elevated levels of anti-tTG IgA (>10 UI/mL) were found in 11 children (5 males and 6 females). Notably, nine of them showed anti-tTG IgA levels 10 times greater than the upper limit of the reference range.

Total serum IgA was measured in all of these 206 patients: 20 children were found to have IgA levels below the age-related reference range and, thus, anti-tTG IgG concentrations were measured. One of these 20 low-IgA children was one of the 2 children finally diagnosed with CD and his anti-tTG IgA levels were not so high as the 10-fold upper normal limit. Notably, this patient showed increased levels of anti-tTG IgG (126.2 UI/mL). No children with complete IgA deficiency were detected in the study population and, except for the aforementioned patient, all showed normal levels of anti-tTG IgG. Unfortunately, as explained in the methods section, the unavailability of the diagnostic kits for EMA did not allow us to skip the biopsy of the duodenal mucosa for the final diagnosis of CD: therefore, all 11 anti-tTG IgA-positive patients underwent upper gastrointestinal endoscopy and received a histopathological diagnosis, according to the Marsh–Oberhuber classification.

In Table 3, we report the main demographic and clinical characteristics of CD patients, which were compared with non-CD children. Overall, the impairment of auxological parameters was more accentuated in CD patients, even if the differences were not statistically significant, probably due to the limited number of CD patients. In terms of gastrointestinal complaints, there is an overlap, except for recurrent abdominal pain, which was reported more frequently in CD children (72.7% vs. 34.9%; *p* = 0.0203). As regards the extra-intestinal manifestations, only anemia and tooth defects were observed more often in CD patients (respectively: 45.5% vs. 15.9%; *p* = 0.0259; 72.7% vs. 31.8%; *p* = 0.0084).

Another interesting finding is provided by the parents’ report of the stool characteristics according to the Bristol scale, as shown in Table 4. Indeed, the occurrence of watery diarrhea (corresponding to type 7) is the only one showing a significant difference between non-CD and CD patients, where it is observed much more frequently (36.3% vs. 6.8%; *p* = 0.0077).

No significant hematological alterations were present, except for lower levels of hemoglobin, based on the age-related reference range; however, in absolute values, no statistically significant differences were found between CD and non-CD children (respectively, 97 [95, 120] vs. 108 [93.7, 114] g/dL; *p* = 0.3287), which is probably due to the different hemoglobin levels according to age. All CD patients had normal values of liver enzymes, except for one, and in general, no significant biochemical abnormalities were observed.

**Figure 1:**
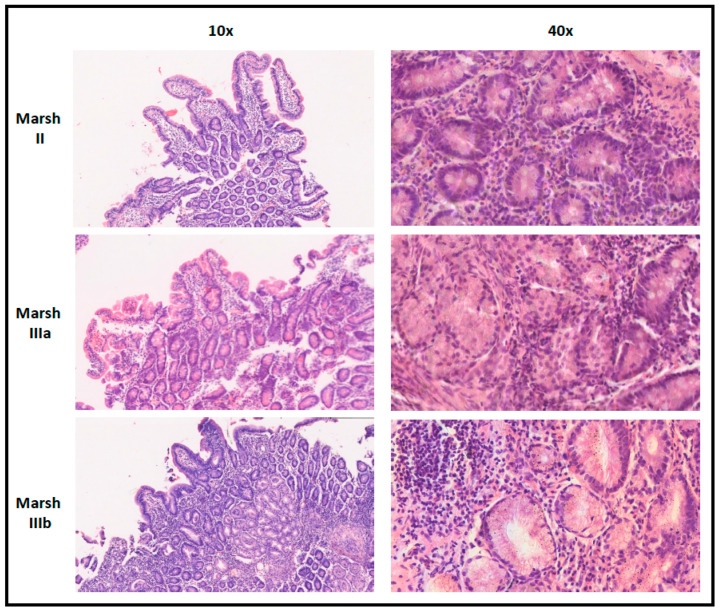
Sample images from these patients for each histopathological pattern according to Marsh grading.

As shown in Table 5, all the CD patients showed anti-tTG IgA 10 times higher than the upper normal limit, except for the patient with low serum levels of total IgA (as described above). As explained, due to the lack of EMA testing, although all CD children were confirmed to carry the HLA-DQ2/DQ8 haplotype, they had to undergo a biopsy of the duodenal mucosa: notably, all children resulted in being histologically positive according to the Marsh histopathological grading (grade II: *n* = 2; grade IIIa: *n* = 2; grade IIIb: *n* = 7; the sample images are shown in Figure 1), which confirms the fact that all these children could have been safely diagnosed without a biopsy according to the current ESPGHAN diagnostic criteria in a setting without resource limitations.

Finally, all these CD children received zonulin and pancreatic elastase measurements on their stools. Six CD patients (54.5%) showed zonulin values above the reference range and an additional two had values in the upper normal range, whereas none showed insufficient levels of pancreatic elastase. No statistically significant correlation or association between zonulin and pancreatic elastase and the parameters reported in Table 5 (and, in general, clinical, laboratory, histopathological, and immunogenetic parameters) was found. In this table, we also reported the CD patients who complained of joint pain (arthralgia): even if 45.5% of them reported this complaint, their frequency of arthralgia was not significantly different from that observed in non-CD children (*n* = 52, 36.4%).

## 4. Discussion

This is the first study investigating the clinical characteristics of pediatric CD in Uzbekistan. In this cohort of 206 symptomatic children, 11 cases of CD were finally ascertained, which corresponds to a prevalence of 5.34% in this very selected (not general) pediatric population. Similar percentages were observed in previous studies from other countries in Europe, Africa, and North America, which are known to have a significant prevalence of CD in the general population; however, most of these studies were not focused on symptomatic patients only and/or also included adults [15,16,17,18].

Anyway, even if we cannot have any information here on the prevalence of CD in the pediatric general population, this initial observation on symptomatic children may provide some indirect and preliminary estimation of pediatric CD burden in Uzbekistan and, more in general, in Central Asia, where specific and reliable information (based on internationally accepted diagnostic criteria) is still missing. Specifically focusing on the prevalence of CD in the pediatric population, in 2014, Cristofori et al. prospectively screened 782 Italian symptomatic children referred to a center specialized in pediatric gastroenterology, as in the present study. Actually, these children were affected by functional gastrointestinal manifestations described as irritable bowel syndrome, dyspepsia, and recurrent abdominal pain; overall, 15 patients finally received a diagnosis of CD confirmed by histology in all these cases (like in the present study), which corresponds to the 1.92% prevalence of CD in this specific population [19]. Of course, this value is lower than ours because, in the present study, we considered a wider spectrum of clinical problems (including chronic diarrhea, recurrent vomiting, constipation, and extra-gastrointestinal manifestations) to apply the CD serological screening, which then could explain this difference due to increased pre-test probability.

However, unlike in Uzbekistan, the prevalence of pediatric CD in Italy has been well estimated and is known to be around 1.5%, if the asymptomatic cases (screened because they belong to at-risk groups) are also included. Notably, these studies screened the pediatric population based on the presence of the HLA-DQ2/-DQ8 CD-predisposing haplotypes [20,21]. A previous study from Central Asia showed that the allelic frequencies of HLA-DQ2/-DQ8 genes in this geographical area are comparable to those observed in European populations [9], which has already suggested the under-diagnosis of CD in Kazakhstan and other neighboring countries. 

Therefore, the significant prevalence of CD that we found among symptomatic Uzbek children, in addition to the aforementioned immunogenetic considerations, suggests that the prevalence of pediatric CD may be similar to that observed in Europe, also considering the relevant consumption of wheat [22,23]. This expectation is further supported by the previous observations of 6–7% anti-tTG IgA seropositivity in at-risk pediatric patients from Kazakhstan [24], and by the fact that significant barriers to CD diagnosis still exist in Central Asia [5], in addition to the previous observations coming from Uzbekistan as well [25,26].

In terms of clinical presentation of CD, no substantial qualitative and/or quantitative differences can be observed compared to the previous studies from different countries [27,28,29]. Notably, we reported an interesting finding related to the assessment of stool consistency according to the Bristol scale [13]. Notably, no significant differences in stool consistency patterns were observed among CD and non-CD children, except for “watery diarrhea with no solid pieces” (type 7), which was much more frequent in the former group. Although “chronic” diarrhea has been traditionally considered as an expression of “classical” CD [30], it is still frequently observed in children with gastrointestinal symptoms who finally receive a diagnosis of CD [28,31,32,33]. Notably, this more severe stool pattern represented by watery diarrhea may be related to the local clinical context which might be characterized by a significant diagnostic delay compared to developed countries. The high Marsh degree (at least III, in >80% of our CD patients) and the presence of very high anti-tTG IgA levels in all our patients, except those with low total IgA levels, may let us speculate about a longer clinical course before CD diagnosis despite such a classical disease presentation, at least in pediatric patients, as also discussed in a couple of recent studies [34,35]. The more accentuated impairment of auxological parameters in our CD patients compared to non-CD children might support this speculation, as discussed by Riznik et al. in their analysis of the clinical presentation of CD children from Central Europe [29]. Conversely, in most developed countries, in children, the clinical pattern of CD has been gradually shifting to a more frequent atypical presentation, where CD presenting with classical gastrointestinal forms is promptly recognized and more attention is paid to extra-gastrointestinal symptoms potentially associated with atypical CD [36,37]. At the same time, these observations further point out the fact that many pediatric patients with CD may not be diagnosed in Uzbekistan due to poor medical awareness and/or diagnostic investigation in children with atypical forms [38], as already reported in other countries of Central Asia [5,24] and in other developing countries [4,39,40,41].

However, some interesting aspects may emerge from this study, especially as regards specific extra-intestinal manifestations in children referred to a pediatric gastroenterologist. In detail, 27.7% of children referred to our center reported recurrent joint pain (without arthritis). Such an association between joint pain (or musculoskeletal pain in general) and gastrointestinal complaints has already been highlighted, especially in patients with functional gastrointestinal disorders, probably due to joint hypermobility in part [42,43]. Nonetheless, well-defined rheumatological conditions are mainly associated with gastrointestinal complaints in patients affected by inflammatory bowel disorders [44,45]. As regards pediatric CD, recent studies have highlighted its increased prevalence in children diagnosed with juvenile idiopathic arthritis [46,47,48]. Interestingly, some previous studies have also reported unspecific musculoskeletal manifestations (including arthralgia) as a frequent complaint in patients later diagnosed with CD [49,50,51]. Even if we found reports of joint pain (arthralgia) in around 45% of our CD patients, we did not find any significant difference in the frequency of this extra-intestinal symptom compared to non-CD children.

Another interesting analysis performed in our CD children was related to the measurement of fecal zonulin, which was found to be increased in around 55% of these patients (*n* = 6) and be in the upper normal range in the other two. Zonulin is a 47-kDa human protein that increases permeability in the epithelial layer of the small intestine by modulating the intercellular tight junctions [51,52,53,54]. Notably, gliadin can stimulate zonulin release from enterocytes and monocytes upon binding the CXCR3 chemokine receptor [54,55]. Although zonulin has been mainly investigated in inflammatory bowel disorders in the last few years [56,57,58,59], this protein complex was initially studied in CD, where tight junctions resulted to be more permeable as a result of zonulin upregulation directly induced by the exposure to the disease antigenic trigger gliadin [52,60,61,62]. Zonulin was also considered for a more general role in the context of immune-mediated or autoimmune manifestations, especially those associated with CD [63,64,65]. In recent years, considering the aforementioned association between CD and chronic arthritis [46,47,48], zonulin was also assessed in this rheumatic clinical context. It was found to be increased in sera and feces of patients affected with rheumatoid arthritis and ankylosing spondylitis [66,67,68]. In the present study, no CD patients were concomitantly diagnosed with juvenile idiopathic arthritis, but almost half of them complained of arthralgia: notably, no statistically significant association between this rheumatological complaint and fecal zonulin was found in these CD patients.

Of course, several limitations affected this study, including access to CD serological screening only for children with prevalent gastrointestinal complaints, the small number of CD patients, and the diagnostic limitations, such as the unavailability of EMA testing. Moreover, the available study design did not allow us to clearly estimate the diagnostic delay in our CD patients and, as regards the data related to fecal zonulin and pancreatic elastase, the research budget constraint was sufficient to perform these analyses in CD patients only (thus without any chance to compare them with non-CD children in this regard). However, this study represents the first prospective attempt to describe the epidemiological burden of pediatric CD in Uzbekistan among children referred to a gastroenterological unit.

## 5. Conclusions

This study may further suggest a significant prevalence of CD among Uzbek children, which is likely to be underestimated. Additionally, we highlight the prevalence of typical forms with watery diarrhea, which in the local clinical setting should strongly support a full diagnostic work-up for CD, since this clinical finding was much more frequent in CD children than in non-CD pediatric patients with gastroenterological complaints. The high levels of anti-tTG IgA and high Marsh grade in our patients might let us speculate about a significant diagnostic delay despite the classical clinical expression, but the available data cannot confirm this aspect, of course. Finally, the analysis of fecal zonulin performed in our CD patients did not show any association and/or correlation with clinical manifestations, general laboratory parameters, histopathological grade, and immunogenetic background.

## Figures and Tables

**Table 1:** Clinical characteristics of the study population.

	*All* *(n = 206)*	*Male* *(n = 98)*	*Female* *(n = 108)*	*p-Value*
* **Gastrointestinal manifestations** *				
***Diarrhea***	72 (34.9%)	36 (36.7%)	36 (33.3%)	0.6091
***Bloating***	173 (84.0%)	80 (81.6%)	93 (86.1%)	0.4483
***Vomiting***	53 (26.1%)	27 (27.5%)	26 (24.1%)	0.6331
***Abdominal pain***	76 (36.8%)	34 (34.7%)	42 (38.9%)	0.5652
***Constipation***	63 (30.6%)	30 (60.6%)	33 (30.5%)	1.0000
* **Extra-gastrointestinal manifestations** *				
***Weight loss***	76 (36.9%)	23 (23.5%)	53 (49.1%)	**0.0002** *
***Growth failure***	50 (24.2%)	19 (19.4%)	31 (28.7%)	0.1437
***Short stature***	85 (41.2%)	35 (35.7%)	50 (46.3%)	0.1564
***Enamel defects***	70 (34.0%)	31 (31.6%)	39 (36.1%)	0.5567
***Anemia***	36 (17.5%)	22 (22.4%)	14 (12.9%)	0.0977
***Headache***	37 (18.0%)	19 (19.4%)	18 (16.7%)	0.7168
***Hyper-transaminasemia***	11 (5.3%)	6 (6.12%)	5 (4.6%)	0.7599
***Oral aphthae***	38 (18.4%)	17 (17.3%)	21 (19.4%)	0.7227
***Delayed puberty***	8 (3.8%)	6 (6.1%)	2 (1.8%)	0.1543
***Joint pain***	57 (27.7%)	27 (27.5%)	30 (27.7%)	1.0000
***Skin rashes***	70 (33.9%)	34 (34.7%)	36 (33.3%)	0.8834
***Auxological parameters [median (IQR)]***				
***Age (years)***	3.6 (2.2, 8.5)	3.6 (1.9, 9.4)	3.5 (2.4, 6.6)	0.2883
***Height (z-score)***	−1.8 (−2.75, −0.71)	−1.94 (−3.14, −0.71)	−1.69 (−2.61, 0.66)	0.4231
***Weight (z-score)***	−1.51 (−2.42, −0.46)	−1.30 (−2.39, 0.23)	−1.66 (−2.48, −0.58)	0.0882
***BMI (z-score)***	−0.60 (−1.57, 0.37)	−0.21 (−1.32, 0.90)	−0.78 (−1.62, 0.01)	**0.0043** *

* Age and auxological parameters are expressed as the median (IQR).

**Table 2:** Clinical characteristics of the study population according to the age classes.

Age Groups	1–4 yrs. (*n* = 112)	5–8 yrs. (*n* = 40)	9–12 yrs. (*n* = 35)	13–17 yrs. (*n* = 19)	*p*-Value
**Gastrointestinal manifestations**					
***Diarrhea***	52 (46.4%)	9 (22.5%)	10 (28.5%)	6 (31.6%)	0.1319
***Bloating***	101 (90.1%)	30 (75.0%)	23 (65.7%)	16 (84.2%)	0.4947
***Vomiting***	34 (30.3%)	9 (22.5%)	8 (22.8%)	9 (47.4%)	0.2232
***Abdominal pain***	36 (32.1%)	14 (35.0%)	13 (37.1%)	13 (68.4%)	0.1207
***Constipation***	38 (33.9%)	14 (35.0%)	9 (25.7%)	4 (21.05%)	0.7139
**Extra-gastrointestinal manifestations**					
***Weight loss***	42 (37.5%)	18 (45.0%)	5 (14.2%)	0 (0%)	**0.0056** *
***Growth failure***	23 (20.5%)	8 (20.0%)	13 (37.1%)	14 (73.7%)	**0.0001** *
***Short stature***	56 (50.0%)	15 (37.5%)	9 (25.7%)	9 (47.3%)	0.2649
***Enamel defects***	35 (31.25%)	16 (40.0%)	15 (37.1%)	12 (63.1%)	0.1426
***Anemia***	23 (20.5%)	4 (10.0%)	6 (17.1%)	8 (42.1%)	0.1028
***Headache***	4 (3.5%)	13 (32.5%)	13 (37.1%)	11 (57.9%)	**0.0001** *
***Hypertransaminasemia***	3 (2.6%)	2 (5.0%)	0 (0%)	1 (5.2%)	0.5724
***Oral aphthae***	22 (19.6%)	2 (5.0%)	10 (28.5%)	8 (42.1%)	**0.0201** *
***Joint pain***	3 (2.7%)	13 (32.5%)	6 (17.1%)	6 (31.6%)	**0.0001** *
***Skin rashes***	20 (17.8%)	8 (20.0%)	10 (28.5%)	7 (36.8%)	0.3172

Abbreviations: yrs., years. * Age and auxological parameters are expressed as the median (IQR).

**Table 3:** Demographic, clinical, and laboratory characteristics of CD children compared to non-CD patients.

	CD Patients (*n* = 11)	Non-CD Patients (*n* = 195)	*p*-Value
**Age (years)**	5.7 (2.0, 13.2)	3.5 (2.2, 8.1)	0.4050
**Gender (M:F)**	5:6	93:102	0.8850
**Height (z-score)**	−2.0 (−3.10, −1.14)	−1.87 (−2.71, −0.64)	0.5787
**Weight (z-score)**	−2.30 (−3.38, −0.96)	−1.45 (−2.29,−0.26)	0.2948
**BMI (z-score)**	−1.15 (−3.44, −0.33)	−0.45 (−1.54, 0.51)	0.0957
**Gastrointestinal manifestations**			
**Diarrhea**	6 (54.5%)	66 (33.8%)	0.2192
**Bloating**	9 (81.8%)	164 (84.1%)	0.6905
**Vomiting**	4 (36.4%)	49 (25.1%)	0.4788
**Abdominal pain**	8 (72.7%)	68 (34.9%)	0.0203 *
**Constipation**	3 (27.3%)	60 (30.8%)	0.3885
**Extra-gastrointestinal** ** manifestation** **s**			
**Weight loss**	6 (54.5%)	70 (35.9%)	0.2192
**Growth failure**	3 (27.3%)	47 (24.1%)	0.7302
**Short stature**	6 (54.5%)	79 (40.5%)	0.3667
**Enamel defects**	8 (72.7%)	62 (31.8%)	0.0084 *
**Anemia**	5 (45.5%)	31 (15.9%)	0.0259 *
**Headache**	2 (18.2%)	35 (17.9%)	1.0000
**Hypertransaminasemia**	2 (18.2%)	8 (4.1%)	0.0923
**Oral aphthae**	3 (27.3%)	35 (17.9%)	0.4295
**Delayed puberty**	1 (9.1%)	7 (3.6%)	0.3604
**Joint pain**	5 (45.5%)	52 (26.7%)	0.1815
**Skin rashes**	2 (18.2%)	68 (34.9%)	0.3390

* Age and auxological parameters are expressed as the median (IQR).

**Table 4:** Stool assessment according to the Bristol scale.

Bristol Stool Scale		*n* = 195	*n* = 11	*p* Value
**Type 1**	Separate hard lumps, like nuts (difficult to pass)	42 (20.4%)	2 (18.2%)	1.0000
**Type 2**	Sausage-shaped, but lumpy	21 (10.2%)	1 (9.1%)	1.0000
**Type 3**	Like a sausage but with cracks on its surface	15 (7.3%)	1 (9.1%)	0.5783
**Type 4**	Like a sausage or snake, smooth and soft (average stool)	52 (25.2%)	0 (0%)	0.0700
**Type 5**	Soft blobs with clear-cut edges	4 (1.9%)	1 (9.1%)	0.2310
**Type 6**	Fluffy pieces with ragged edges, a mushy stool (diarrhea)	58 (28.2%)	2 (18.2%)	0.7313
**Type 7**	Watery, no solid pieces, entirely liquid (watery diarrhea)	14 (6.8%)	4 (36.3%)	**0.0077** *

* Age and auxological parameters are expressed as the median (IQR).

**Table 5:** Specific diagnostic work-up in CD patients.

Patients (n)	Anti-tTG IgA (U/mL)	Total IgA (mg/dL)	HLA-DQA1 (2 alleles)	HLA-DQB1 (2 alleles)	Marsh Grade	Zonulin (15–107 ng/mL)	Elastase (>200 mcg/mL)	Joint Pain
**#1**	110	2760	0301*0201	0302*0601	IIIb	104.5	685	-
**#2**	131.2	200	0101*0501	0201*0602	IIIb	35	420	Y
**#3**	171.8	350	0201*0501	0302*0303	IIIb	47.5	268.1	-
**#4**	20.5	30	0201*0501	0201*0201	IIIa	224.1	360	Y
**#5**	134.8	226	0103*0501	0201*0201	II	180.1	723.6	-
**#6**	141.2	366	0301*0501	0303*0302	IIIb	60.9	231.5	Y
**#7**	429.4	310	0201*0501	0503*0201	IIIb	160.7	814	-
**#8**	109	140	0201*0501	0201*0601	IIIa	200.1	440	Y
**#9**	203.2	210	0103*0501	0503*0201	IIIb	81.6	620	-
**#10**	105.3	223	0201*0101	0201*0501	IIIb	199	341.7	-
**#11**	155	157	0301*0103	0302*0303	II	310.1	150.1	Y

## Data Availability

The data presented in this study are available from the corresponding author upon request. The data are not publicly available due to confidentiality reasons.
